# Novel Hemostatic Technique During Laparoscopic Liver Parenchymal Transection: Saline-Linked Electrocautery Combined With Wet Oxidized Cellulose (SLiC-WOC) Method

**DOI:** 10.7759/cureus.27431

**Published:** 2022-07-29

**Authors:** Yusuke Uemoto, Takahisa Fujikawa, Yusuke Kawamoto, Masatoshi Kajiwara

**Affiliations:** 1 Surgery, Kokura Memorial Hospital, Kitakyushu, JPN; 2 Surgery, Tokyo Women's Medical University, Tokyo, JPN; 3 Gastroenterological Surgery, Faculty of Medicine, Fukuoka University, Fukuoka, JPN

**Keywords:** low-temperature heat coagulation, saline-linked electrocautery, oxidized cellulose, liver parenchymal transection, laparoscopic liver resection

## Abstract

Introduction: Although laparoscopic hepatectomy has the potential advantage of reducing intraoperative blood loss, it is more difficult to control bleeding laparoscopically compared to an open approach. We introduced a novel hemostatic technique, the saline-linked electrocautery combined with wet oxidized cellulose (SLiC-WOC) method, during laparoscopic hepatectomy where a combination of saline-linked electrocautery (SLiC) and wet oxidized cellulose (WOC) is used. This study aimed to investigate the feasibility of employing the SLiC-WOC method for laparoscopic hepatectomy.

Methods: Thirteen patients who underwent laparoscopic liver resection with the SLiC-WOC method between 2019 and 2020 were included in this study. The number of bleeding episodes in which the SLiC-WOC method was applied was counted, and the time required to achieve complete hemostasis was measured.

Results: Among the bleeding events that were difficult to achieve hemostasis by SLiC alone, 94% were safely and efficiently controlled. Additionally, 69% of hemostasis was achieved within 60 seconds and 91% within 120 seconds. Postoperatively, most patients experienced no complications and no operative mortality was observed.

Conclusions: The SLiC-WOC method can provide safe and time-efficient hemostasis during laparoscopic hepatectomy. This is especially crucial for bleeding, which is difficult to control using electrocautery alone.

## Introduction

Laparoscopic liver resection (LLR) is believed to offer potential advantages over open surgery in relation to intraoperative bleeding and transfusion [1,2]. However, the laparoscopic method is more challenging to manage compared to the open approach if bleeding occurs. Expanding the indication of LLR for patients with cirrhosis or those who are receiving preoperative antithrombotic therapy for thromboembolism may also lead to an increased risk of intraoperative bleeding, even though it has been demonstrated that lowering airway pressure or maintaining low central venous pressure reduces hepatic vein bleeding [3-5].

Most intraoperative blood loss during liver resection occurs during transection of the liver parenchyma. Numerous hemostatic techniques have been examined as supplemental techniques for liver resection. With varying degrees of efficacy, topical hemostatic treatments such as oxidized cellulose [6], microfibrillar collagen [7], and human thrombin and fibrinogen patches [8] have been employed for hemostasis. Saline-linked electrocautery (SLiC) has also been developed as a valuable tool for liver resection hemostasis [9-11]. Nevertheless, complete hemostasis is not always achieved when using these substances or tools alone.

Recently, we proposed the saline-linked electrocautery combined with wet oxidized cellulose (SLiC-WOC) method, a novel technique that merges SLiC with wet oxidized cellulose (WOC) [12]. During LLR, this procedure can offer hemostasis for bleeding, which is difficult to stop using traditional approaches. In this study, we examine the application of this technique and review its effectiveness and safety.

## Materials and methods

Between February 2019 and May 2020, 24 patients underwent conventional LLR at our institution. Patients with large liver tumors with diameters greater than 10 cm as well as those being considered for vascular reconstruction or multi-visceral resection are generally excluded from LLR. However, in all other cases, such as patients with liver cirrhosis, cardiovascular and/or pulmonary comorbidity, or prior abdominal operations, liver resections are mainly performed laparoscopically.

Nonanatomical and anatomical hepatectomies were performed in 15 and nine patients, respectively. The SLiC-WOC method was applied to 13 patients. The number of bleeding episodes in which the SLiC-WOC method was applied was counted, and the time required to achieve complete hemostasis was measured. The study protocol complied with the Declaration of Helsinki and was approved by Kokura Memorial Hospital Clinical Research Ethics Committee (# 15062403). The requirement for written informed consent was waived owing to the retrospective nature of the study.

Surgical technique

Using a 12-mm umbilical trocar, pneumoperitoneum was established and maintained at 8-10 mmHg. Four functioning trocars were positioned in accordance with the tumor location, and a laparoscope was inserted via the umbilical trocar. For right or left lobe tumors, a 5-mm incision in the left or right mid-upper abdomen was made to establish a tourniquet for the Pringle technique. The Pringle technique was used when necessary, and the central venous pressure was kept as low as feasible to limit bleeding during parenchymal transection [3,13].

We established and maintained a "modified two-surgeon approach" for liver parenchymal transection during LLR [11] based on the "Kyoto University-style liver parenchymal transection" procedure [13] during open liver resection. Briefly, the primary surgeon uses a Cavitron ultrasonic surgical aspirator (CUSA; Valleylab, Boulder, Colorado) or ultrasonic coagulating shears (CS; Ethicon, Cincinnati, Ohio) to dissect the hepatic parenchyma from the patient's right side. Intraparenchymal vessels smaller than 2 mm are dissected using a CS. Vascular structures with a diameter greater than 2 mm are divided by clipping or ligating them with 3-0 polypropylene ties. In the meantime, the secondary surgeon focuses on creating hemostasis while holding a ball-tipped SLiC and suction tube in each hand. The soft coagulation mode is set to effect 5 and 70 W when the ball-tipped SLiC is plugged into an electrosurgical VIO device (ERBE Elektromedizin GmbH, Tübingen, Germany). The irrigation tube is linked to a sterile 0.9% saline bottle (1 L), and the drip rate is set at 1-2 cc/minute.

**Figure 1:**
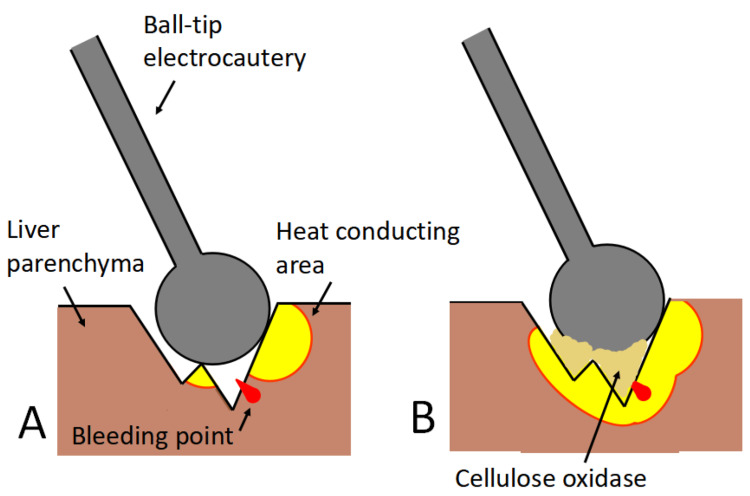
The mechanism of hemostasis using the saline-linked electrocautery combined with wet oxidized cellulose (SLiC-WOC) method. (A) Bleeding from the bottom of the parenchymal fissure in which direct access to the bleeding point is difficult by application of the SLiC alone. (B) By interposing WOC between the SLiC and the bleeding site, heat from the SLiC is transmitted effectively via the WOC to the bleeding point in the deep parenchymal fissure, allowing hemostasis to be achieved. SLiC: saline-linked electrocautery; WOC: wet oxidized cellulose.

**Figure 2:**
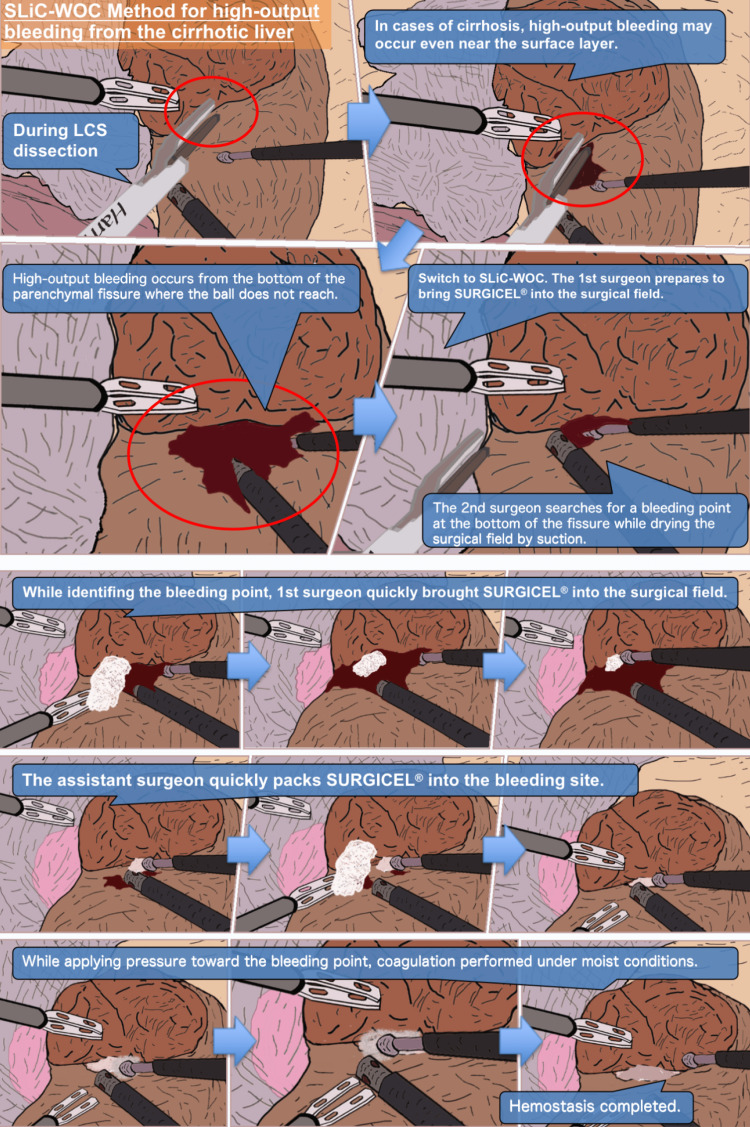
The process of hemostasis via the SLiC-WOC method in the case of high-output bleeding from the cirrhotic liver. SLiC-WOC: saline-linked electrocautery combined with wet oxidized cellulose; LCS: laparoscopic coagulating shears.

**Figure 3:**
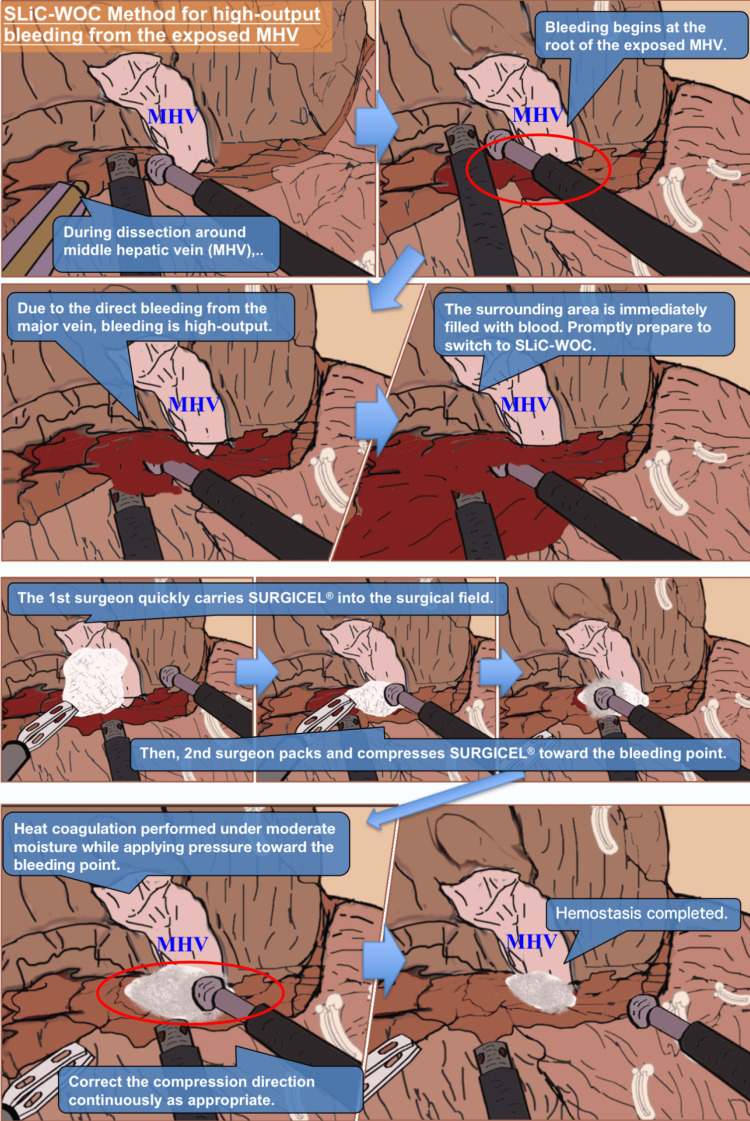
The process of hemostasis via the SLiC-WOC method in the case of high-output bleeding from the exposed middle hepatic vein. SLiC-WOC: saline-linked electrocautery combined with wet oxidized cellulose; MHV: middle hepatic vein.

Hemostasis with the SLiC is initially performed when bleeding from the dissected parenchyma occurs. However, it is challenging to control bleeding from the bottom of the cut surface or from the small cavity in the dissected parenchyma. The SLiC-WOC method is used if hemostasis is not achieved after 20 seconds or if it is anticipated to be difficult to achieve using the SLiC alone, such as bleeding from thick vessels (Figure 1). Figures 2, 3 and Video 1 depict the general framework of the SLiC-WOC method for high-output bleeding.

**Video 1:** The outline of the hemostatic process via the SLiC-WOC method (with narration). This video provides a summary of the SLiC-WOC method used during laparoscopic liver resection. SLiC-WOC: saline-linked electrocautery combined with wet oxidized cellulose.

The primary surgeon quickly applies the fibrillar type of oxidized cellulose (SURGICEL® FIBRILLAR^TM^ Absorbable Hemostat, Ethicon, Cincinnati, Ohio) to the surgical field and stretches the field when high-output bleeding occurs. The secondary surgeon uses a suction tube to search for a bleeding point, while the primary surgeon quickly stretches the surgical field. A sufficient amount of SURGICEL® is packed into the bleeding area by the secondary surgeon, who also coordinates the use of the SLiC and suction tube. Saline solution is drip-fed from the SLiC onto the implanted SURGICEL® to saturate it. Excess saline solution and blood are continually suctioned away from the bleeding area to prevent accumulation. Finally, hemostasis is quickly achieved by compressing and activating the SLiC toward the bleeding spot through the saline-soaked SURGICEL® (Video 2). To avoid heating the bile duct, which may result in bile leakage or bile duct stenosis, the SLiC should be compressed in the direction of the counterpart of the branch when the bleeding site is near a branch of the Glissonean pedicle.

**Video 2:** The SLiC-WOC method for high-output bleeding during laparoscopic liver resection. This video provides an overview of the SLiC-WOC method for high-output bleeding from a cirrhotic liver or exposed middle hepatic vein. Reproduced from reference [11], with permission. SLiC-WOC: saline-linked electrocautery combined with wet oxidized cellulose.

## Results

Among the 13 patients included in the study, no conversion to open hepatectomy was required. The median operative time was 249 (157-614) minutes, with a median blood loss of 60 (0-1520) mL. None of the patients required perioperative blood transfusion. Details of the 13 patients are shown in Table 1.

**Table 1:** Patient characteristics. AP: angina pectoris; ACT: anticoagulation therapy; APT: antiplatelet therapy; ATT: antithrombic therapy; CoCC: cholangiocellular carcinoma; DAPT: dual antiplatelet therapy; DM: diabetes mellitus; F: female; HCC: hepatocellular carcinoma; M: male; Meta: metastatic tumor; PE: pulmonary embolism; SLiC-WOC: saline-linked electrocautery combined with wet oxidized cellulose.

No.	Sex/age (years)	Diagnosis	Procedure	Comorbidities	ATT	Liver status	Operative time (minutes)	Blood loss (ml)	Number of bleeding SLiC-WOC is applied	Number of hemostasis achieved by SLiC-WOC
1	M/73	HCC	Partial	Obesity, AP, DM	None	Cirrhotic	307	60	1	1
2	M/82	HCC	Right posterior sectionectomy	Obesity, DM	None	Normal	549	230	3	3
3	M/57	Meta	Partial	DM	None	Cirrhotic	176	35	3	3
4	F/44	Angiomyolipoma	Partial	None	None	Normal	259	80	3	3
5	M/67	HCC	Partial	DM	None	Cirrhotic	249	70	1	1
6	M/72	HCC	Partial	None	None	Cirrhotic	175	35	1	1
7	F/66	Meta	Partial	AP	APT	Normal	157	60	1	1
8	M/70	HCC	Segmentectomy 5	PE	ACT	Cirrhotic	250	60	4	4
9	M/85	HCC	Segmentectomy 5	None	DAPT	Cirrhotic	236	45	1	1
10	F/51	CoCC	Left medial sectionectomy	Obesity, AP	None	Cirrhotic	614	1520	8	6
11	M/69	HCC	Partial	AP	None	Normal	174	0	1	1
12	M/78	HCC	Left hepatectomy	None	None	Cirrhotic	448	110	2	2
13	F/75	Meta	Lateral sectionectomy	DM	None	Normal	200	40	3	3

**Figure 4:**
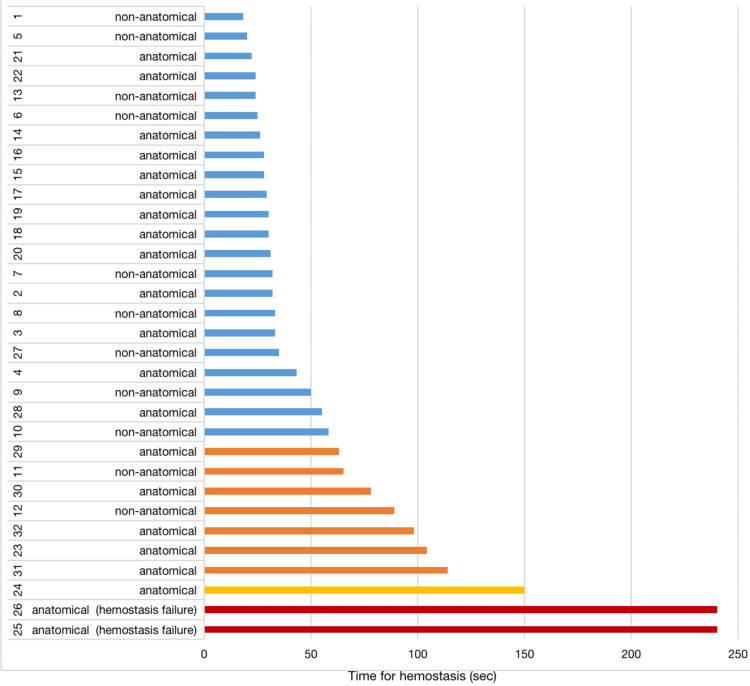
Swimmer plot of 32 hemostatic procedures using the SLiC-WOC method for intractable bleeding during LLR. SLiC-WOC: saline-linked electrocautery combined with wet oxidized cellulose; LLR: laparoscopic liver resection.

**Figure 5:**
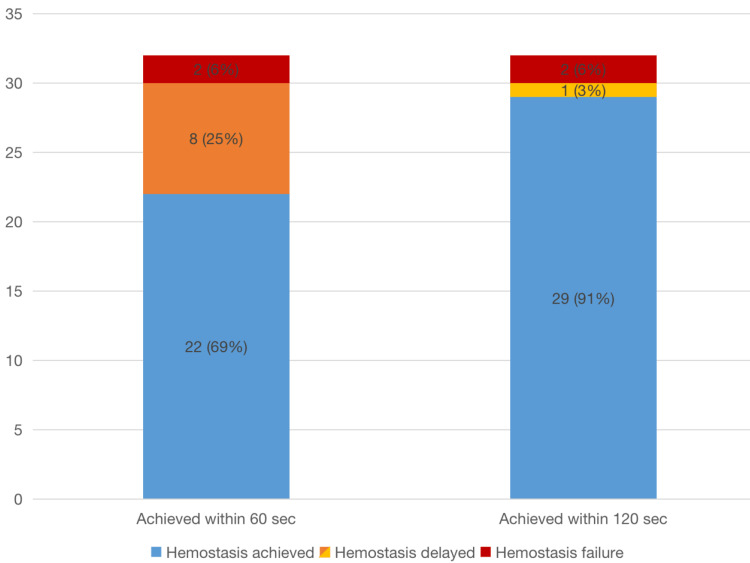
Rates of hemostasis achieved within 60 and 120 seconds using the SLiC-WOC method during laparoscopic liver parenchymal transection. SLiC-WOC: saline-linked electrocautery combined with wet oxidized cellulose.

The SLiC-WOC method was applied 32 times, and hemostasis was achieved 30 times (94%; Figure 4). As seen in Figure 5, the median time for hemostasis in 30 cases was 33 (18-150) seconds. Furthermore, 69% of hemostasis was achieved within 60 seconds, and 91% within 120 seconds (Figure 5). Two areas of bleeding could not be controlled using the SLiC-WOC method. The first bleeding originated from the middle hepatic vein stump on the resected side of the liver, which was dissected using the CS alone. The second bleeding was from the stump of the Glissonean branch of segment four on the resected side of the liver, which was also dissected using CS alone.

Most patients experienced no complications postoperatively, except for one patient with an intra-abdominal abscess (4.2%), which was resolved conservatively. No postoperative mortality was observed.

## Discussion

The current study demonstrates that the SLiC-WOC method accomplishes hemostasis against uncontrollable bleeding from the bottom of the cut surface or the small cavity during LLR with a high probability (94%). Moreover, it may be performed without the risk of bile leakage or bile duct blockage as a result of heat damage. Therefore, the SLiC-WOC technique can deliver safe and time-effective hemostasis during LLR.

It is crucial to perform laparoscopy in a dry (blood-free) surgical field with a magnified field of vision. Controlling pneumoperitoneum pressure, central venous pressure, and airway pressure, performing the Pringle maneuver, and employing an energy device are all ways to reduce bleeding in LLR [3-5,14-17]. The introduction of a soft coagulation device (VIO) to achieve hemostasis during liver dissection is particularly ground-breaking [17]. These techniques significantly reduce bleeding in liver transection. However, it may be challenging to stop bleeding from veins or Glisson's capsule at the bottom of a deep parenchymal fissure, even when using a soft coagulation device.

Electrocautery, sutures, and compression with gauze or hemostatic substances are the three methods used to stop bleeding. Owing to restrictions on the mobility of the tool and the use of bi-dimensional vision, suturing is one of the most challenging procedures in laparoscopic surgery. Although compression at the bleeding site is simple to perform and effective in stopping bleeding, it takes a few minutes to complete the task and not all bleeding can be halted. Gauze compression can temporarily produce hemostasis, but blood pools make it difficult to see the surgical area. Furthermore, a blood clot may detach prematurely and reinitiate the bleeding. Finally, gauze, which is less flexible than other topical treatments, may not stick to the tissue surface and convey sufficient heat to the bleeding site.

In contrast to the methods mentioned above or the SLiC alone, the SLiC-WOC approach combines two different hemostatic techniques: WOC compression and SLiC low-temperature coagulation [12]. This results in efficient and reliable hemostasis. Another advantage of the SLiC-WOC approach is the protection of bile ducts and deep parenchyma from heat damage through the use of low temperatures. When electrocautery is activated using a WOC, the ball tip is cooled, and the surface temperature is maintained at or below 100°C without making direct contact with the hepatic tissue. During the observation period of the study, no postoperative biliary leakage was detected. Oxidized cellulose is a rayon produced by the oxidation of plant-derived cellulose fibers. The qualities of rayon include flexibility and water compatibility. Primary hemostasis is facilitated by the creation of a coagulum through salt generation and a strong affinity between oxidized cellulose fibers and hemoglobin. Efficient compression of the bleeding site is obtained because the flexible oxidized cellulose fits the tissue around the bleeding point.

The production of eschar and char, which is related to rebleeding [18], is also avoided by interposing the WOC. Theoretically, the SLiC alone can prevent eschar and char formation at the tip of the electrocautery. However, in the clinical setting, insufficient amounts of saline solution and/or pooling of blood can occasionally result in the electrocautery coming into direct contact with the hepatic tissue, which results in the formation of char or eschar. Complete hemostasis is possible by placing the WOC between the SLiC and the bleeding site because heat from the SLiC is efficiently conveyed by the WOC even to the bottom of the cut surface or the tiny space in the dissected parenchyma where direct access by the SLiC alone is impossible (Figure 2).

This study has some limitations. Due to the retrospective nature, it only partially predicts how treatments affected the results. Furthermore, the sample size was small, and a larger sample size would probably be helpful for producing more reliable suggestions. Finally, a control group is required for the comparison of our novel technique to a conventional procedure. Although advanced LLR is not a currently commonly selected surgical procedure, we believe that the SLiC-WOC approach is a step toward the future standardization of laparoscopic liver parenchymal transection. This may not only result in a safer and more efficient LLR but also permit its adoption on a larger scale.

## Conclusions

The SLiC-WOC method promotes smooth surgical progress since it is safe and efficient for hemostasis during liver transection. With this technique, the majority of uncontrollable bleeding from the liver parenchymal fissure can be promptly managed. The SLiC-WOC method can be utilized even for bleeding, which is challenging to control using electrocautery alone.
